# Opiate agonist treatment to improve health of individuals with opioid use disorder in Lebanon

**DOI:** 10.1186/s12954-017-0204-8

**Published:** 2017-12-08

**Authors:** Ali Ghaddar, Zeinab Abbas, Ramzi Haddad

**Affiliations:** 1 Department of Biomedical Sciences, Lebanese International University, Beirut, Lebanon; 2 Observatory of Public Policies and Health, Beirut, Lebanon; 3 School of Pharmacy, Lebanese International University, Beirut, Lebanon; 4 Department of Narcotics, Ministry of Public Health, Beirut, Lebanon; 5 Department of Psychiatry, Lebanese University, Beirut, Lebanon; 6 Department of Psychiatry, Saint Joseph University, Beirut, Lebanon

**Keywords:** Buprenorphine, Harm reduction, Opiate-related disorders, Lebanon

## Abstract

**Background:**

Opioid agonist therapy has been widely used to reduce harms among individuals with opioid use disorder but its effectiveness has not been evaluated in the Middle East North African (MENA) region. This study aims to evaluate the effectiveness of a program using opioid agonist therapy in combination with psychosocial support on improving psychological and social well-being, reducing arrest, and reducing risky behavior in individuals with opioid use disorder in Lebanon.

**Methods:**

A one-group pre-test post-test design study was performed at SKOUN Lebanese Addiction Centre between January 2013 and December 2014. Eighty-six out of 181 patients agreed to participate and completed the 3-month assessment and 38 concluded the 12-month assessment. Psychological (depression and anxiety, quality of life), substance dependence/abuse, behavioral (injecting behavior, sharing needles and paraphernalia), and social outcomes were evaluated at baseline, 3, and 12 months post-treatment.

**Results:**

Remarkable statistical significance improvements were observed 3 months after treatment in most outcome variables including quality of life, anxiety, substance dependence, overdose, employment, and injecting behavior. Improvements were sustained 12 months after treatment.

**Conclusion:**

Results support expanding the access to opioid agonist therapy in other MENA countries to treat substance dependence and reduce harms among individuals with opioid use disorder.

## Background

Opioid agonist treatment (OAT) has become the main treatment approach for people with opioid dependency and a fundamental component of the evidence-based harm reduction approach to HIV prevention in many developed countries [1]. Its implementation has resulted in a marked reduction in HIV-related risky behavior including injecting needles and sharing syringes leading to a decline in HIV transmission, HIV incidence, and mortality associated with unsafe injection [2, 3] and in preventing the spread of hepatitis C virus among injecting drug users (IDUs) [4]. OAT has been increasingly used in order to decrease the health, economic, and social consequences of substance abuse and to improve quality of life (QoL) of opioid-dependent users [5–8].

OAT involves the provision of opioid agonist such as methadone and buprenorphine. The choice of the drug and of the modality of its provision (observed vs. take-home) influences treatment outcomes, yet there is little consensus about the most effective treatment regime [9]. A synthesis of evidence obtained from placebo-controlled trials revealed that buprenorphine is effective in suppressing illicit opioid use [9] and in (at higher doses) retaining patients in treatment although to a lesser extent than methadone [10, 11]. On the other hand, buprenorphine has longer duration and limited withdrawal syndrome, and patients who receive buprenorphine were more likely to test negative for opioid use compared to those receiving methadone [12]. It also has a safer profile over methadone in terms of reducing mortality [11] and reducing diversion-related death [13]. In general, international guidelines emphasize direct observational induction of buprenorphine followed by multiple in-clinic visits [14]. However, with less strict supervision, unobserved take-home induction supposes fewer logistic barriers and thus results in better treatment outcomes including prolonged abstinence from opioids and reduced drug use [15, 16].

Despite its proven effectiveness and affordability, OAT is still unavailable in many low- and middle-income countries where it is desperately needed [1]. In such countries, evidence from a WHO collaborative study suggested that OAT reduced illegal opioid use, HIV-associated risk behaviors, and criminality, and substantially improved physical and mental health among opioid-dependent patients [17]. OAT also resulted in substantial improvements in QoL and in several domains of Addiction Severity Index including drug use, psychological well-being, criminality, and family relations [18]. OAT also demonstrated positive impact on QoL and physical, psychological, and social well-being among opioid users in Malaysia, Lithuania [19, 20], and Ukraine [21] and on reducing injecting behavior, criminality, HIV infections, and improving QoL in China [22–24]. Similarly, in Taiwan, OAT showed significant improvements in health-related QoL, psychological, and social well-being, and HIV-related risky behavior [25]. Indeed, there is increasing evidence suggesting that OAT could attain consistent outcomes in a culturally diverse range of settings in both low- and middle-income and high-income countries. In another study, OAT significantly reduced arrest incidences, risky behavior, and improved QoL [26]. Likewise, other studies encouraged to scale up OAT in low- and middle-income countries to save lives and resources [2]. This issue is of particular importance as the epidemic of dependence on prescription opioids is predicted to spread to low- and middle-income countries [27].

So far, OAT has been provided in at least five Middle East and North African (MENA) countries. Morocco developed a national plan that enabled the introduction of methadone substation treatment pilot programs in 2010. Bahrain, United Arab Emirates, and Palestine have also recently started providing OAT. Iran adopted OAT in its national policy and is the pioneer and leader in outpatient programs with estimated 4275 dispensing centers. In MENA countries, OAT program outcomes have been evaluated only in Iran where evidence support its effectiveness in reducing drug use, injection behavior, and syringe sharing and in improving health, QoL [28], and social well-being among IDUs [29].

In 2011, Lebanon adopted an OAT take-home buprenorphine pilot program only provided by authorized psychiatrists working within pre-registered treatment settings. One particularity of the treatment modality in Lebanon is the provision of psychosocial support as a basic component of the treatment. After being assessed for eligibility to treatment (diagnosis with opioid use disorder according to DSM5 criteria), patients are followed on weekly basis by a multidisciplinary team consisting of a psychiatrist, psychologist, social worker, and registered nurse. During follow-up, patients are monitored through regular urine tests for opiates, buprenorphine, and other drugs and are assessed for possible misuse, diversion, stability, and response to treatment. Furthermore, it is worth to mention that in Lebanon, a national study conducted in 2010 showed that heroin use accounted for 50% of patients treated for addiction and described a high rate of relapse among heroin users. It also revealed a high rate of arrest related to heroin use, with half of the treated patients having been already arrested at least once by the police [30]. Another particularity of OAT in Lebanon is that it could be a way out of arrest related to drug use, as drug use is criminalized in Lebanon except for users registered in treatment programs.

Almost 6 years has passed since the program initiation in Lebanon in 2011, yet, there exist no published reports about its effectiveness. Furthermore, the effectiveness of an OAT approach combining the component of psychosocial support provided by multidisciplinary team has not been well explored in previous research. The current study aims to evaluate the effectiveness of the pilot OAT program in Lebanon implemented by multidisciplinary teams on treating substance use disorders, improving the mental health and social functioning, and in reducing risky behavior among individuals with opioid use disorder. It also aims to explore the program outcome on reducing arrest related to drug use among registered users.

## Methods

### Assessment

The Lebanese Public Health Ministry (MOPH) implemented buprenorphine for treating opioid use disorder as evidence documents its feasibility, comparable safety profile, and fewer logistic barriers than observed induction [14]. The national guidelines specify that patients receive weekly take-home buprenorphine prescribed by authorized psychiatrists working within pre-registered treatment settings. Eligible patients diagnosed with opioid use disorder according to DSM5 or ICD10 (WHO) criteria are followed up on weekly basis by multidisciplinary teams consisting of a psychiatrist, psychologist, social worker, and registered nurse. Patients are monitored through regular urine tests for opioids and buprenorphine and are assessed for possible misuse, stability, and response to treatment during regular visits to treatment center.

Upon admission, participants completed a self-administered questionnaire that included variables on socio-demographic characteristics, medical history, and substance use. Participants were interviewed by trained psychologists that were part of the treatment team and were trained for the purpose of the project. For each patient, questionnaires were filled systematically at three time intervals: upon admission (baseline), 3, and 12 months post-treatment.

The research protocol was approved by the institutional review boards at SKOUN and at the Lebanese International University. A one-group pre-test post-test design was adopted in order to measure the effect of OAT by examining the difference in the outcomes at baseline, 3, and 12 months post-treatment.

### Participants

SKOUN Lebanese Addiction Center is the first and largest outpatient community-based treatment center to implement OAT in Lebanon and currently enrolls around 40% of the OAT patients in Lebanon. Participants in the study were opioid-dependent patients who sought treatment at SKOUN. During the study period (January 2013–December 2014), patients diagnosed with opioid use disorder according to the Diagnostic and Statistical Manual of Mental Disorders (DSM5) who were prescribed OAT at SKOUN were approached for participation (181 male patients). Women were not included as there is a very low prevalence of women seeking treatment, especially for heroin dependence. Participants were clearly explained that refusal to participate will not affect their treatment process. Eighty-six patients agreed to participate and concluded the 3-month follow-up assessment, out of which 38 concluded the 12-month follow-up assessment. Participants were briefed by the team members about the objectives of the research and signed a written informed consent prior to participation. Patients were explained that refusal to participate will not affect positively or negatively their treatment. Ethical approval was obtained from the Institutional Review Board of the Lebanese International University.

### Measures

Participants were asked about their age, educational level, average household income, marital status, employment criminal activity, number of arrests, and number of days in prison. Several participants had along with heroin dependence other substance dependence (cocaine and cannabis). We monitored the outcome of OAT on reducing heroin dependence along with cocaine and cannabis dependence. Substance use disorder (i.e., heroin, cocaine, and cannabis) was assessed according to DSM5 criteria (American Psychiatric Association, 2000).

Self-reports concerning participants’ general health in the last 2 weeks were measured using EUROHIS QoL Scale (WHO QOL-8) validated questionnaire [31]. The scales were composed of eight questions with Likert-type scale with five response options (1 = very poor, 5 = very good). The utilized scales were translated and blindly back translated in Arabic by two separate professional translators. An expert panel helped solve discrepancies between both translations and the final version was used.

The Hospital Anxiety and Depression Scale (HADS) was used to assess psychological well-being [32]. Each of anxiety and depression was measured by seven items with Likert-type scale with four response options (0 = not at all, 3 = most of the times). Anxiety was assessed by asking whether participants experienced tense or frightened feelings, worrying thoughts, or whether they felt restless. Depression was assessed through questions such as “I have lost interest in my appearance.” The score of each dimension is established by the summation of the individual questions. Cronbach’s alpha reliability coefficients of the scales in the sample were 0.77, 0.84, and 0.81 for QoL, anxiety, and depression, respectively.

Injecting behavior was measured through a scale from 1 to 4 (1 = during last 3 months and 4 = never) and sharing needles was measured through a scale from 1 to 6 (1 = more than 10 times and 6 = never) based on a questionnaire that measures risk for transmission of blood-borne viruses [33]. Overdose was measured through a simple question about overdose during last 3 months.

### Statistical analysis

We displayed in the results only the pretreatment variables that explained at least 1% of the variance in our health outcomes (Table 1). The other pre-treatment variables that could not account for variance in the outcome variables (eta-square less than 0.01) are not mentioned in the table. Shapiro-Wilk test was used to check the normality of distribution of the outcome variables. For continuous variables that showed normal distribution (QoL, anxiety, and depression), within-group changes were analyzed using paired, one-tailed *t* test (for the 3-month assessment) and one-way repeated measure ANOVA (for the 12-month assessment). For the other variables that were not normally distributed, changes in nominal categorical variables (previous arrest, work) were analyzed using the McNemar non-parametric tests with effect sizes calculated in accordance with Cohen’s *d*. Differences in ordinal variables (injecting behavior and sharing needles) were analyzed by *z*-Wilcoxon rank non-parametric tests with calculating *r*-correlation coefficient for effect size. *α*-level of 5% was considered statistically significant.

**Table 1:** Pre-treatment individual characteristics of participants who completed the 3-month assessment (*n* = 86) and the 12-month assessment (*n* = 38)

	3 months	12 months
Age, years	*n* = 86	*n =* 38
18–25	32 (37.2%)	15 (39.5%)
26–30	25 (29.1%)	11 (28.9%)
31–35	20 (23.3%)	9 (23.7%)
36+	9 (10.5%)	3 (7.9%)
Educational level	*n* = 84	*n =* 37
Illiterate	19 (22.6%)	5 (13.5%)
School	44 (52.4%)	19 (51.4%)
University	21 (25.0%)	13 (35.1%)
Average household income, $	*n* = 77	*n =* 34
Low (< 1000)	43 (50.0%)	20 (52.6%)
Middle (1000–2500)	25 (29.1%)	11 (28.9%)
High (2500+)	9 (10.5%)	3 (7.9%)
Marital status	*n* = 86	*n =* 38
Never married	60 (69.8%)	24 (63.2%)
Ever married	26 (30.2%)	14 (36.8%)
Number of times arrested	*n* = 86	*n =* 38
None	24 (27.9%)	10 (26.3%)
1	20 (23.3%)	11 (28.9%)
≥ 2	42 (48.8%)	17 (44.7%)
Longest duration of detainment, days	*n* = 86	*n =* 38
0	23 (26.7%)	10 (26.3%)
1–30	21 (24.4%)	12 (31.6%)
≥ 1 month	42 (48.8%)	16 (42.1%)
Substance concern	*n* = 86	*n =* 38
Heroine	76 (89.4%)	36 (94.7%)
Cocaine	10 (11.6%)	6 (15.8%)
Cannabis	15 (17.4%)	7 (18.4%)

## Results

### Sample characteristics

The baseline characteristics of participants who completed the 3- and the 12-month assessment are displayed in Table 1. Participants age ranged between 18 and 66 years with median = 28 years (standard deviation = 7.61). Around 73% of participants had history of arrest. The number of previous arrests ranged between 0 and 18 times with median = 2 times (standard deviation = 2.86). Participants stayed in prison between 0 and 6330 days with median = 20 days (standard deviation = 716.27 days). Around half of the participants had school education and had low average household income, and the majority were never married.

Results indicated that the treatment had positive effects on the quality of life, anxiety, and depression that were sustained over the two post-treatment time intervals. QoL scores significantly increased while anxiety scores significantly decreased 3 and 12 months after the treatment. Likewise, depression scores dropped after the treatment, although the difference in scores compared to baseline was statistically significant at the 3-month interval but lost significance at the 12-month interval. However, although reductions in anxiety scores were maintained 12 months after the treatment, the difference between baseline scores of anxiety lost statistical significance (Table 2).

**Table 2:** Changes in mean (s.d.) in outcomes 3 and 12 months post-treatment

	3-month assessment (*n* = 75)				12-month assessment (*n* = 36)				
Variable and group	Baseline	Post 3 months	*p* value	Effect size	Baseline	Post 3 months	Post 12 months	*p* value	Effect size
EUROHIS Quality of Life Scale (8–40) mean (s.d.)	21.82 (6.59)	24.93 (6.32)	*p* ≤ 0.001	Cohen’s d = 0.37	22.37 (6.22)	24.16 (5.82)	24.84 (6.92)	*p* = 0.05	η 2 = 0.14
Anxiety (HADS) (0–21) mean (s.d.)	9.62 (4.09)	7.29 (4.55)	*p* = 0.001	Cohen’s d = 0.34	9.27 (3.84)	7.18 (4.19)	6.91 (3.91)	*p* = 0.006	η 2 = 0.24
Depression (HADS) (0–21) mean (s.d.)	8.97 (3.56)	7.66 (3.69)	*p* = 0.03	Cohen’s d = 0.21	8.94 (3.58)	8.01 (3.54)	7.65 (3.82)	*p* = 0.19	η 2 = 0.17

*HADS* Hospital Anxiety Depression, *s.d.* standard deviation, *d* Cohen’s *d* effect size, *η 2* eta-squared effect size

The treatment had positive effects on reducing arrest and improving employment over time. McNemar’s test determined that there was a statistically significant reduction in the proportion of persons arrested, at the two time intervals (3 and 12 months) post-treatment compared to baseline. However, the % of employed patients increased 3 months after treatment (statistically significant difference) and 12 months after treatment (non-significant difference). Similarly, the McNemar test showed statistically significant reductions in the % of patients who reported overdosing and the % of patients who met heroin, cocaine, and cannabis dependence criteria, at both the 3 and 12 months’ time intervals after the treatment (Table 3).

**Table 3:** Changes in frequency and % in outcomes 3 and 12 months post-treatment

	*n* (%)	*p* value
Arrested last 3 months—3-month assessment (*n =* 86)		
Baseline	62 (72.1%)	
3 months	7 (8.1%)	0.001
Arrested last 3 months—12-month assessment (*n =* 38)		
Baseline	24 (70.6%)	
12 months	1 (2.6%)	0.001
Working—3-month assessment (*n =* 86)		
Baseline	24 (27.9%)	
3 months	52 (60.5%)	0.02
Working—12-month assessment (*n =* 38)		
Baseline	12 (31.6%)	0.07
12 months	30 (78.9%)	
*N* times overdose—3-month assessment (*n =* 86)		
Baseline	12 (14.0%)	
3 months	3 (3.5%)*	0.02
*N* times overdose—12-month assessment (*n =* 38)		
Baseline	8 (21.1%)	
12 months	1 (2.6%)	0.03
Heroin use disorder—3-month assessment (*n =* 86)		
Baseline	72 (100%)	
3 months	5 (6.9%)	≤ 0.001
Heroin use disorder—12-month assessment (*n =* 38)		
Baseline	38 (100%)	
12 months	1 (2.6%)	≤ 0.001
Cocaine use disorder—3-month assessment (*n =* 86)		
Baseline	13 (18%)	
3 months	1 (1.3%)	*p* = 0.04
Cocaine use disorder—12-month assessment (*n =* 38)		
Baseline	6 (15.8%)	
12 months	1 (2.6%)	*p* = 0.01
Marijuana use disorder—3-month assessment (*n =* 86)		
Baseline	18 (25%)	
3 months	6 (9.7%)	*p* = 0.01
Marijuana use disorder—12-month assessment (*n =* 38)		
Baseline	8 (21%)	
12 months	3 (7.8%)	0.04

Results of the Wilcoxon *z*-non-parametric test indicated a slight, statistically non-significant reduction in injecting behavior scores at the 3-month interval (*z* = − 1.23; *p* value = 0.15; effect size *r* = 0.09) and the 12-month intervals (*z* = − 0.56; *p* value = 0.21; effect size *r* = 0.07). Similarly, the Wilcoxon *z* test showed significant reduction in the score of sharing needles 3 months (*z* = − 1.67; *p* value = 0.001; effect size *r* = 0.12) and non-significant reduction in the scores 12 months after the treatment (*z* = − 0.22; *p* value = 0.82; effect size *r* = 0.03). Fewer patients reported overdosing after 3 and 12 months compared to baseline. Significant reductions were noted in the % of patients who met opioid use disorder, cocaine use disorder, and cannabis use disorder 3 and 12 months after treatment.

## Discussion

The primary outcome evaluation of OAT pilot program in Lebanon supports its implementation for treating substance use disorder and reducing harms among individuals with opioid use disorder. Remarkable statistically significant improvements were observed over time (3 and 12 months) among patients under buprenorphine in most outcome variables including QoL, anxiety, substance dependence, overdose, and employment. Congruent findings have been observed in low- and middle-income countries about the effectiveness of buprenorphine in improving mental health and in reducing risky behaviors [17].

The most impressive finding was improvement in employment, not something usually noted in evaluations performed in high-income countries. Statistically significant improvements in QoL were documented after treatment, in consistence with previous findings in low- and middle-income countries [18]. Contrary to findings obtained in other countries, the percentage of arrest among patients receiving OST significantly decreased after the treatment [34]. A possible explanation of this discrepancy in the results is that participants were arrested due to drug use, which is considered a criminal act in Lebanon. Registering in the OAT program is a way out of penalizing persons using drugs. This probably explains the high number of arrest reported before engaging in the program and the dramatic drop 3 months after treatment.

### Limitations

The current study has some limitations related to the study population and design. The absence of a control group and of randomization poses limitations to internal validity related to the difficulty to exclude confounders. The sample size was not calculated a priori, was relatively small, and represented exclusively male heroin-dependent users. Finally, results are prone to information bias related to self-reporting as reported improvement could have been possibility overestimated. Actually, it could be argued that patients usually enter treatment at a time of crisis, when social functioning is poor and self-report is prone to overstate poor health. On the other hand, there is a selection bias related to non-response and loss to follow-up, as one could argue that those followed up are the ones who were successful and that only 47.5% concluded the 3-month assessment. A possible explanation for patient non-participation and dropout could be the transfer of patients to other centers or due to patients’ own will to stop treatment. A more comprehensive evaluation is needed to monitor the benefits of the treatment on a longer term.

## Conclusion

Results have important implications to guide policy makers in making informed decisions about treatment options of individuals with opioid use disorder. The challenges related to the difficult socio-political context that faced the implementation of OAT program in Lebanon since its launching in December 2011 should be taken into consideration [35]. Other MENA countries implementing OAT should also take into consideration the specificities of the treatment protocol adopted by the Lebanese pilot program and should address adopting alternative treatment provision protocols. For instance, in the Lebanese example, a positive evaluation of retention in the program was given to patients who tested negative in the weekly urine test. Following the latest evidence about using more frequent urine testing would allow for a more objective evaluation of the retention in the program and provide better outcomes on sustaining opioid-free urinalysis [36]. Further evaluations of the effectiveness of OAT countries are warranted to monitor the potential adverse effects associated with buprenorphine misuse in MENA [1, 37]. The encouraging results of the evaluation of the first pilot OAT program in Lebanon support expanding the access to buprenorphine in Lebanon and other MENA countries in order to treat individuals with opioid use disorder.

## Abbreviations

HIV: Human immunodeficiency virus
IDUs: Injecting drug users
MENA: Middle East North Africa
MoH: Ministry of Public Health
OAT: Opioid agonist therapy
QoL: Quality of life
